# Full Squats Enhance Performance and Body Composition, but Not Hypertrophy, Compared to Half Squats in Elite Young Tennis Players

**DOI:** 10.3390/jfmk10040440

**Published:** 2025-11-13

**Authors:** Raouf Hammami, Agustín Jerez-Martínez, Pablo Jiménez-Martínez, Carlos Alix-Fages, Haithem Rebai, Oussema Kassis, Álvaro Juesas, Carlos Balsalobre-Fernández, Juan C. Colado, Javier Gene-Morales

**Affiliations:** 1 Higher Institute of Sport and Physical Education of Ksar-Said, Manouba University, Manouba 2010, Tunisia; raouf.cnmss@gmail.com (R.H.); haithem.rebai@isseps.usf.tn (H.R.); kassis.oussama@gmail.com (O.K.); 2 Tunisian Research Laboratory ‘Sports Performance Optimization’ (CNMSS-LR09SEP01), National Center of Medicine and Science in Sports (CNMSS), Tunis 1003, Tunisia; 3 Faculty of Sport Sciences, Catholic University of Murcia (UCAM), 30107 Murcia, Spain; ajerez.training@gmail.com; 4 Department of Health Research, ICEN Canary University, 38010 Santa Cruz, Spain; pablowlfit@gmail.com (P.J.-M.); carlosalixentrenamientos@gmail.com (C.A.-F.); 5 Research Group in Prevention and Health in Exercise and Sport (PHES), Department of Physical Education and Sports, University of Valencia, 46010 Valencia, Spain; alvaro.juesastorres@uchceu.es (Á.J.); juan.colado@uv.es (J.C.C.); javier.gene@uv.es (J.G.-M.); 6 Applied Biomechanics and Sports Technology Research Group, Department of Physical Education, Sport, and Human Movement, Autonomous University of Madrid, 28049 Madrid, Spain; 7 Department of Education Sciences, CEU Cardenal Herrera University, 12006 Castellon de la Plana, Spain

**Keywords:** resistance training, squat, youth athletes, strength conditioning, training specificity

## Abstract

**Background**: The aim was to compare the effects of full squat (FST) versus half squat training (HST) on body composition, muscle hypertrophy, and mean propulsive velocity (MPV) in young athletes. **Methods**: Twenty-eight highly trained male tennis players (13.88 ± 0.91 years, 166.08 ± 11.30 cm, 57.40 ± 8.99 kg, 14.34 ± 2.75% body fat) were randomly allocated to an eight-week FST or HST program. Training volume load was matched between interventions, and the only difference was the range of motion (squat depth). Pre- and post-training tests evaluated body composition (body mass and body fat percent), muscle hypertrophy (muscle volume of the thigh, calf, and leg, and cross-sectional area at half and maximum circumference of the thigh), and MPV at 45 and 50% of one-repetition maximum (1RM). An analysis of variance was used to analyze differences. **Results**: The results exhibited significant group-by-time interactions for body mass (*p* = 0.002, ηp^2^ = 0.32), body fat (*p* < 0.001, ηp^2^ = 0.71), and MPV (all *p* ≤ 0.005, ηp^2^ ≥ 0.27). Post hoc comparisons showed that both groups presented significant improvements in body composition, muscle hypertrophy, and MPV (all *p* ≤ 0.004). However, FST outperformed HST in body fat (*p* = 0.032) and MPV at both %1RM (*p* < 0.001). **Conclusions**: Overall, FST would be preferred over HST for tennis training in youth athletes. Four to five sets of 8–12 repetitions at 60–70% 1RM, two days a week during preseason, appear to be sufficient to induce neuromuscular performance improvement and enhance body composition.

## 1. Introduction

The back squat is a foundational exercise within resistance training (RT), it strengthens the lower limb and enhances athletic performance [1]. The back squat provides notable improvements in both functional and specific performance domains across diverse disciplines, such as rowing [1], soccer [2], cycling [3], and table tennis [4,5]. Moreover, there is a direct relationship between muscle size and strength increases and a lower injury rate [6]. Squat protocols induce distinct physiological responses, contingent upon the strategic manipulation of training load variables, including exercise selection, volume, intensity, velocity of execution, and range of motion [7]. The main adaptations occur as a consequence of intricate neuromuscular and biochemical processes, encompassing heightened motor unit recruitment, synchronization, and firing frequency, alongside augmented myofibrillar protein synthesis and activation of satellite cells [8]. These processes collectively contribute to enhanced force generation and an increase in muscle fiber cross-sectional area [9].

Several squat variations exist in terms of range of motion, including the full squat, parallel squat, and partial squat [10]. The parallel squat concludes the eccentric phase when the inguinal fold aligns horizontally with the top of the knee [11]. Angles above this threshold are classified as partial squats; for instance, the half squat extends to 90° of knee flexion. Full squats are performed until the posterior thighs and calves make contact [11]. These depth variations influence biomechanical and physiological aspects related to force development, motor unit activation and synchronization, and dynamic joint stability [11]. Traditionally, training at partial depths, such as the half or quarter squat, has been recommended based on specificity to certain sports like running or jumping [12]. Conversely, deeper squats are associated with RT modalities like weightlifting, powerlifting, or bodybuilding [10]. Recent research suggests that prolonged RT involving deeper squats may enhance neuromuscular function and athletic performance while mitigating injury risk to passive tissues compared to shorter ranges of motion [13,14]. Hence, achieving optimal squat depth is crucial for maximizing muscle activation, enhancing motor control, and ultimately, reducing the likelihood of injury while enhancing athletic progress.

There is limited literature assessing the impact of squat depth on muscle size. Kubo et al. [15] suggested that a 10-week full squat training (FST) elicits superior effects in lower limb muscle development, notably targeting the adductor and gluteus maximus, compared to half squat training (HST). Additionally, only three studies have explored the impact of varying squat depths in periodized RT regimens of 10–16 weeks on neuromuscular and functional adaptations [12,13,14]. Findings from these studies consistently favored greater strength gains (i.e., 1RM and mean propulsive velocity) with full squats compared to partial squats. Hartmann et al. [13] and Pallarés et al. [14] reported improvements across performance assessments, including the Wingate test, countermovement jump, squat jump, and timed 0–20 m test, favoring full squats over half squats. Conversely, Rhea et al. [12] documented superior performance enhancements in the squat jump and 40-yard sprint with quarter squats. Considering the biomechanical differences based on squat depth [11], the findings underscore that squat depth significantly influences the load–velocity and load–power relationships across the three analyzed squat variations. Due to these conflicting findings and the scarcity of specific studies addressing the various neuromuscular, functional, and anthropometric adaptations associated with different squat depths, further research is warranted to establish robust recommendations for strength and conditioning coaches and the general population.

Therefore, the present study aimed to compare mean propulsive velocity (MPV), body composition, and muscle volume changes between FST and HST in elite male youth tennis players. This information may provide valuable insights for coaches and sports scientists regarding which squat depth optimizes neuromuscular and anthropometric adaptations to achieve desired performance outcomes. We hypothesized that FST would provide the best results in all the variables analyzed.

## 2. Materials and Methods

### 2.1. Study Design

A randomized controlled trial was used to examine the effects of FST vs. HST on measures of body composition (body mass and body fat percentage), muscle volume, and MPV at 45 and 50% of 1RM in highly trained youth tennis players. The training programs were conducted during the preseason period of 2023 (from September to November) and lasted eight weeks, consisting of two weekly FST or HST sessions implemented following the warm-up. Training volumes were matched between groups. Based on previously published training studies [16,17], a true control group could not be incorporated as both experimental groups were national-level elite players, and there were no comparable players available who would provide similar baseline values. Baseline measurements were performed four days after the familiarization session and included body composition (body mass and body fat percentage), muscle volume, and MPV at 45 and 50% of 1RM.

### 2.2. Participants

The sample size estimation was computed using G*Power software (version 3.1.6). Based on findings from a related study [14] examining the effects of full versus partial squat training on mean propulsive velocity in sedentary men, an a priori power analysis with a type I error of 0.05, 95% statistical power, and effect size (f[V]) for the significant full squat results of 0.72 was computed. The analysis indicated that 28 players would be needed to observe significant interaction effects. Accordingly, a total of 28 highly trained male youth tennis players from the same tennis club were recruited to participate in this study.

Twenty-eight male tennis players were randomly (https://www.random.org) assigned to FST (n = 14) or HST (n = 14) by external staff not involved in data collection or training. Researchers in charge of data collection were blinded to group allocation. All participants had practiced systematic tennis training for at least 4–5 years before the study participation. Of note, all groups followed the same tennis training program under the supervision of the same coaches. Participants’ biological maturity status was estimated based on the maturity offset method using the prediction equation of Moore et al. [18].

Before the start of the study, athletes received a document detailing the experimental procedures alongside a parental consent request form. Parental consent and participant assent were obtained after a thorough explanation of the study’s purpose, procedures, risks, and benefits. The present study was conducted following the latest version of the Declaration of Helsinki, and the protocol was fully approved by the Local Ethics Committee of the National Centre of Medicine and Science of Sports of Tunis (CNMSS-LR09SEP01) before the commencement of the procedure. None of the participating players had a history of psychological and musculoskeletal, neurological, or orthopedic disorders six months before the start of the study.

### 2.3. Procedures

One week before the start of the study, a familiarization session was scheduled to allow players to become acquainted with the applied tests and exercises. Participants were asked to keep their usual nutrition and rest habits and were instructed on the proper techniques specific to the FST and HST. The same test sequence was used during pre- and post-tests. More specifically, athletes underwent: (a) body composition evaluation, (b) muscle hypertrophy assessment, and (c) MPV tests at 45 and 50% during the full and half squat 1RM tests. Before testing, all participants conducted a standardized 10 min warm-up which consisted of submaximal running (e.g., skipping, hip in and out), balance exercises (e.g., forward and backward beam walking, single-leg stance on unstable devices), and neuromuscular activation (e.g., dynamic and isometric squat, one leg deep squat). All tests were separated by a 5–10 min rest period. The best out of two attempts was used for further statistical analyses. The rest between each attempt was three minutes.

### 2.4. Anthropometrics

Athletes’ body height and mass were collected using a wall-mounted stadiometer (OHAUS, Florham Park, NJ, USA) and an electronic scale (Baty International, West Sussex, UK), respectively. The sum of skinfolds was assessed using skinfold calipers (Baty International, West Sussex, UK). Body measurements were conducted according to Deurenberg et al. [19] who reported similar prediction errors between adults and adolescents. Afterward, biological maturity was evaluated non-invasively using chronological age and body height as input parameters for a regression equation to subsequently predict the maturity offset. The equation has previously been validated for boys and presents a standard error of estimate reported as 0.542 years [18]. The measurements showed excellent absolute and relative reliability for both, body mass and body fat percentage, with ICC values of ≥0.943, and CV ≤ 1.17%.

#### 2.4.1. Leg Muscle Volume

Muscle volume can be calculated using geometric approximations based on anthropometric measurements (e.g., circumference and length). The leg refers to the entire lower limb, including thigh and calf, and its muscle volume is the sum of the thigh and calf volumes in cm^3^ [20]. For the thigh volume, we should approximate the thigh as a truncated cone using the following formula:

$$\mathrm{Volume}=\frac{\pi h}{12}(C_{1}^{2}+C_{1}C_{2}+C_{2}^{2})$$

where:

• *h* = height/length of the thigh (measured from hip to knee joint).
• *C*₁ = circumference at the proximal end (near the hip).
• *C*₂ = circumference at the distal end (near the knee).

Measurements of the maximum calf circumference and just above the ankle circumference and skinfolds on the back and each side of the calf plus leg length (from the trochanter major to the lateral malleolus) were used to calculate the calf muscle volume in cm^3^ [20]. Calf length is often assessed using a flexible measuring tape, ensuring the participant is in a relaxed, standing position with weight evenly distributed. The measurement is taken from the lateral malleolus (outer ankle bone) to the popliteal fossa, following the natural contour of the leg [9].

The reliability for muscle volume measurements of the leg, thigh, and calf are generally high, indicating good reliability with ICC ranging between 0.850 and 0.980 and a CV between 1.50 and 5.00% [21].

#### 2.4.2. Cross-Sectional Area (CSA) of the Thigh

The thigh CSA was calculated from mid-thigh and maximal circumference of the thigh [22]. Considering the circumference of the thigh as a circle, its radius R was calculated as:

Circumference (C) = 2π × Radius (R); therefore: R = C/2π

The radius of the muscular component of the mid-thigh (r) was estimated by removing the double thickness of anterior and posterior skin folds to the total radius:

r = R − [(mid-thigh anterior skin fold + mid-thigh posterior skin fold)/4]

Finally, the thigh CSA equaled π × r^2^ (cm^2^).

### 2.5. Neuromuscular Performance

Following the familiarization sessions, the individual load–velocity relationships were determined by means of a progressive loading test up to the 1RM for the FST and HST. Following the warm-up, initial load was set at 20 kg and was gradually increased in 10–15 kg increments until the attained mean propulsive velocity (MPV) was ≤0.60 m/s [23]. Thereafter, load was individually adjusted with smaller increments (5 down to 2.5 kg) so that the 1RM could be precisely determined. Three repetitions were executed for light loads (>0.60 m/s) and one for heavy loads (≤0.60 m/s). The 1RM was considered as the heaviest load that each subject could properly lift while completing full ROM for each squat, without external help. Inter-set recoveries ranged from 3 min (light loads) to 5 min (heavy loads). A very high test–retest reliability of this testing protocol (ICC = 0.99, 95% CI = 0.99–1.00, CV = 2.5%) has been recently described [24].

For the FST and HST, stance width and feet position were individually adjusted and carefully replicated on every lift. Participants started from an upright position, with the knees and hips fully extended, stance approximately shoulder-width apart with both feet flat on the floor and parallel or externally rotated to a maximum of 15°. The barbell rested on the back at the level of the acromion. From this position, they were required to descend in a continuous motion until reaching their previously determined concentric initial position for each squat variation: HST: descent until reaching a 90° knee angle [13]. FST: descent until the first of these two criteria was met: (i) when posterior thighs and calves made contact with each other, or (ii) when the lumbar spine angle was equal to 180° [11]. Participants were required to perform the concentric phase explosively (at maximal intended velocity) and the eccentric phase at a controlled mean velocity of 0.45–0.65 m/s [25]. Repetitions that failed to meet any of these requirements were automatically discarded and repeated after a 3 min rest. All testing and training lifts were made using a Smith machine with no counterweight mechanism.

Participants were required to perform the concentric phase explosively (at maximal intended velocity) and the eccentric phase at a controlled mean velocity of 0.45–0.65 m s^−1^ [25]. This protocol was practiced during the familiarization sessions conducted. Repetitions that failed to meet any of these requirements were automatically discarded and repeated after a 3 min rest. Measures from the following neuromuscular parameters were considered for the analysis: 1RM strength in kg, average MPV attained against all absolute loads common to T0 and T1 (MPVALL), average MPV attained against absolute loads lower than 50% 1RM common to T0 and T1 (MPV absolute loads higher than 50% 1RM common to T0 and T1 (MPV > 50% 1RM, “high” loads).

### 2.6. Training Program

The intervention consisted of an 8-week, 2 days per week squat RT program. As it has been indicated [26,27], when prescribing training volume for the learning stage of RT in young athletes, multiple sets and lower repetitions are most effective to learn the squat, using a 5–10 kg youth-sized weightlifting bar with wooden plates. Therefore, a beginner may be prescribed 1–5 sets of 8–12 repetitions with a light or moderate load (60–70% 1RM or equivalent) [27]. The two experimental groups (FST and HST) trained with the same relative loading magnitude (progressively increasing from 60% to 70% 1RM over the time course of the study), inter-set recoveries (4 min), and volume (4–5 sets and 8–12 repetitions) but differed in the depth of the squat trained (Table 1). During training, participants received immediate velocity feedback while being encouraged to perform each repetition at the maximal intended velocity. Participants were required to perform the concentric phase explosively (at maximal intended velocity) and the eccentric phase at a controlled mean velocity of 0.45–0.65 m·s^−1^ [25]. This procedure ensures that each athlete performs every squat repetition at the programmed load intensity during the training sessions, thus avoiding the mismatches that typically occur when programming is solely based on the % 1RM value measured in T0 [14]. For both groups, participants started from 60% 1 RM during weeks 1 and 2, 70% 1RM during weeks 3 and 4, 60% 1 RM during weeks 5 and 6, and 70% 1RM during weeks 7 and 8. As recently pointed out, 60% 1 RM, the 1RM value measured at the beginning of a RT program (T0) will be considerably altered over the training weeks due to the neuromuscular improvements and/or fatigue processes that occur in the athlete’s functional performance [25]. At the start of the intervention, a rating of perceived exertion (RPE) of 3 on the OMNI Perceived Exertion Scale for Resistance Exercise (OMNI-RES) was targeted. During weeks 3–4, the RPE was increased to 5, RPE of 7 for weeks 5 and 6, and, finally, in weeks 7–8, a RPE of 9 was targeted. Training volume load (i.e., total amount of work, repetitions x relative load) was equal between the two groups. Qualified coaches and experienced sports scientists supervised the intervention groups. A standardized warm-up including jogging, dynamic stretching exercises, calisthenics, and preparatory exercises (e.g., fundamental weightlifting exercises specific to their training program) was provided for all experimental groups before the beginning of each training session. Each training session ended with 5 min of cool-down activities, including dynamic stretching.

### 2.7. Statistical Analyses

All the statistical tests were conducted using SPSS for Macintosh (version 28.0, IBM Corp, Armonk, NY, USA). Results are reported as mean, standard deviation, and 95% confidence interval (CI). The significance level was set at *p* < 0.05.

After basic data curation, the normality of data distribution was checked with the Shapiro–Wilk test. All variables showed a normal Gaussian distribution (*p* > 0.05) except the calf muscle volume and cross-sectional areas in the FST Group, the leg muscle volume in both groups. However, all the variables complied with the sphericity assumption with Mauchly’s W test and presented homogeneous variances. Therefore, a two-way mixed analysis of variance (ANOVA) of repeated measures was conducted considering it is largely robust to violations of normality when the sphericity assumption is met [28]. The ANOVA evaluated the effects of the time (pre-intervention, post-intervention) and the training group (FST vs. HST) as the within and between-participants factors, respectively. The effect size was calculated through the eta partial squared (ηp^2^), where 0.01 < ηp^2^ < 0.06 constitutes a small effect, 0.06 ≤ ηp^2^ ≤ 0.14 medium, and ηp^2^ > 0.14 a large effect. Afterward, we conducted Bonferroni-adjusted post hoc comparisons. The effect size was calculated with SPSS as Cohen’s d (based on the corrected standard deviation of the difference) and interpreted as trivial (<0.20), small (0.20–0.49), moderate (0.50–0.79), or large effects (≥0.80).

The test–retest relative reliability of the instruments was assessed by calculating the ICC (model: two-way mixed, type: consistency) [29]. ICC was interpreted as poor (<0.40), moderate (0.40–0.59), good (0.60–0.79), or excellent (≥0.80) [30]. The absolute reliability was verified with the coefficient of variation (CV) using the formula: (standard error of measurement (SEM)/mean of both measurements)x100; SEM is the standard deviation of the difference between the two measurements divided by the square root of the number of measurements per subject [31]. It was defined as excellent reproducibility with a CV ≤ 10%, good reproducibility with a CV between 10 and 20%, acceptable with a CV between 20 and 30%, and poor reproducibility with a CV > 30% [32].

## 3. Results

### 3.1. Participants

**Figure 1:**
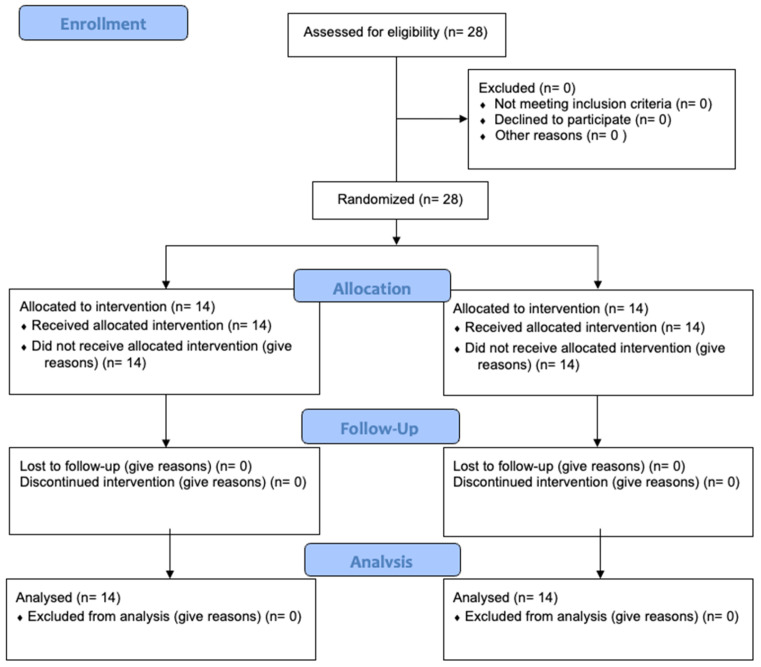
CONSORT Flow Diagram.

All the 28 young athletes that started the study completed all the procedures (Figure 1). Participants attended all testing sessions, and none reported any training- or test-related injury. The adherence rate was 100% for all participating athletes. There were no significant differences between groups in any of the descriptive features of the sample (all *p* > 0.05). The specific values for each participants' feature are presented inTable 2

### 3.2. Anthropometry and Body Composition

There was a significant effect of time in all the anthropometry and body composition variables (all *p* < 0.001, ηp^2^ ≥ 0.46). On the other hand, the interaction time*group was only significant for body mass (*p* = 0.002, ηp^2^ = 0.32) and body fat percentage (*p* < 0.001, ηp^2^ = 0.71). The post hoc comparisons showed that both groups presented significant improvements (reductions in body fat and increases in body mass, muscle volume, and cross-sectional area) in all the studied variables (all *p* < 0.001) except the FST Group in their body mass (*p* = 0.403). However, there were only significant between-group differences in the body fat percentage at the post-intervention measurements (*p* = 0.032), with better values (less body fat) for the FST Group (Table 3).

### 3.3. Neuromuscular Performance

There was a significant effect of time and time*group in all the neuromuscular performance variables (all *p* ≤ 0.005, ηp^2^ ≥ 0.27). The post hoc comparisons showed that both groups presented significant increases in MPV (all *p* ≤ 0.004). Additionally, while there were non-significant between-group differences in the pre-intervention measurements (all *p* ≥ 0.087), highly significant between-group differences existed in the post-intervention measurements (all *p* < 0.001), with better values (higher MPV) for the FST Group (Table 4).

**Figure 2:**
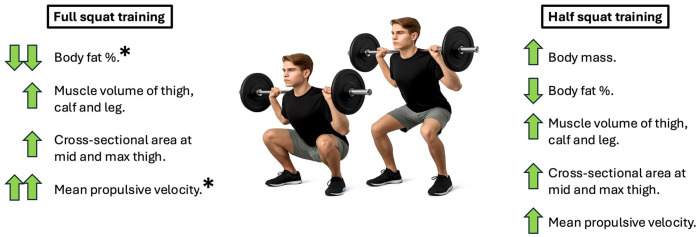
Summary of the study outcomes. “*” denotes significant between-group differences, single arrow represents increment or decrement without statistical significance between groups and double arrows indicate the group with better values.

Figure 2 presents a summary of the outcomes.

## 4. Discussion

This study aimed to compare the effects of FST and HST protocols over 8 weeks on MPV, body composition, and muscle hypertrophy among elite male youth tennis players. These outcomes serve as a valuable resource for designing RT regimens aimed at enhancing athletic performance and body composition.

The principal findings of the present study revealed that the FST elicited notable enhancements in MPV at 45 and 50% 1RM and reductions in body fat percentage compared to the HST protocol. Additionally, no statistically significant between-group differences were observed in other anthropometric variables such as body mass, lower-limb muscle volume, and CSA. These results underscore the efficacy of FST in fostering superior neuromuscular adaptations and optimizing body composition among elite youth athletes. Furthermore, both FST and HST were equally effective in promoting hypertrophy of the thigh and calf musculature.

The existing evidence supports the assertion that training at deeper ranges of motion yields the most substantial neuromuscular strength adaptations [13,14,33]. Conversely, Rhea et al. [12] documented superior performance enhancements in the vertical jump and 40-yard sprint with quarter squats over FST. These divergent outcomes may stem from methodological disparities in the training protocols employed across studies. For instance, Rhea et al. [12] included supplementary exercises beyond squats (e.g., power cleans, lunges, reverse hamstring curls, and step-ups) without regulating the range of motion, which potentially confounded the results. In our study, both FST and HST demonstrated enhancements across all neuromuscular variables (MPV at 50 and 45% 1RM), with statistically significant superior outcomes for the FST. Regarding muscle hypertrophy, our results can only be compared to the study conducted by Kubo et al. [15], wherein a 10-week protocol involving physically active young men revealed no significant between-group differences in knee extensor muscle volume. Thus, these findings align with our own. However, Kubo et al. [15] did observe greater volume enhancement for the FST compared to the HST in the adductor and gluteus maximus, which were not evaluated in our study.

Therefore, considering the outcomes of this study and requirements for elite young athletes to enhance athletic performance while minimizing injury risk, it is imperative to assess which depth of squatting entails lower risks of knee injury and pain. Hartmann et al. [13] compared half and quarter squats against full squats, concluding that full squats induce reduced stress on the knee and spinal joints. Similarly, Pallarés et al. [14] assessed pain perception and physical functional disability at different squat depths, noting higher levels in half squats compared to parallel and full squats, contrary to popular beliefs. This suggests that shallow depths may yield a suboptimal mechanical force position during the concentric phase while lifting a greater weight load at the same relative load (% 1RM), with up to a 1.5-fold increase in half squats compared to full squats at 80% 1RM [11] or even double at 60–80% 1RM [14].

In this study, we employed several methodological adjustments to ensure the robustness and effectiveness of our research protocol. Initially, we conducted a comprehensive assessment of individual load–velocity relationships through a progressive loading test, extending up to the 1RM for both FST and HST groups. This meticulous approach facilitated precise and personalized load adjustments based on mean propulsive velocity, thereby optimizing the training. Immediate velocity feedback was provided during training sessions to ensure compliance with prescribed velocity targets and enhance training efficacy [14]. Additionally, to further individualize the training load, we implemented a systematic RPE scale to regulate training intensity throughout the intervention period [34]. This strategy aimed to optimize the training stimulus while minimizing the risk of both overexertion and under-training [34].

Our study also yielded significant insights into anthropometric changes post-intervention, showing marked improvements across all variables, including reduced body fat percentage and increased muscle volume and CSA in both training groups. However, FST demonstrated significantly greater reductions in body fat percentage, suggesting a potential advantage of this approach in body composition management. One possible explanation for this phenomenon may be the increased time under tension during each repetition in FST compared to HST, which leads to higher training volume, increases glycolysis metabolism, and may promote greater muscle adaptations by stimulating delayed muscle protein synthesis at 24–30 h of recovery [35]. These findings hold practical implications for training strategies, particularly in the context of young athletes. The observed improvements in body composition, characterized by reduced body fat and increased muscle volume, suggest that squats, especially full squats, can effectively enhance body composition parameters in this population. Optimizing body composition is crucial for performance and injury prevention in tennis [5], where rapid changes in direction and landings induce heightened joint stress, especially in the lower extremities. Our results underscore the potential utility of FST in promoting favorable changes in body composition, thereby contributing to enhanced athletic performance and injury prevention among young athletes [36,37]. Conversely, investigations examining the influence of squat depth on body composition parameters such as weight and body fat percentage are scarce and require further exploration.

### 4.1. Limitations and Future Directions

Future research to cover the study limitations could entail the application of this intervention protocol across diverse demographics, encompassing adults, elderly individuals, and sex-specific analysis to elucidate potential disparities in neuromuscular and anthropometric adaptations. While our study sample consisted of rather experienced youth tennis players, the findings of this study should be compared with those of novice players. It also would be interesting to compare the adaptations to squat protocols performed on Smith machine or with free weights. These comparisons would advance comprehension regarding the effects of distinct squat depths on diverse adaptations, thereby expanding the applicability of optimized training methodologies across different populations.

### 4.2. Practical Applications

As practical applications from a practitioner’s point of view, FST should be preferred over HST for tennis training in youth athletes. Four to five sets of 8–12 repetitions at 60–70% 1RM, two days a week during preseason, appear to be sufficient to induce neuromuscular performance improvement and enhance body composition. However, HST could be more appropriate for less experienced tennis players due to the safer nature of this squat variation for beginners.

## 5. Conclusions

In conclusion, FST demonstrates greater effectiveness in improving neuromuscular performance and reducing body fat percentage compared to HST in elite male youth tennis players. These findings highlight the importance of full squat variations to enhance physical performance in athletic populations. Using FST as in the current paper is, therefore, an effective tool for coaches and practitioners to enhance in-season neuromuscular adaptation and body composition in young tennis players.

## Figures and Tables

**Table 1:** Design of the 8-week full and half squat training program.

Weeks	Week 1	Week 2	Week 3	Week 4	Week 5	Week 6	Week 7	Week 8
%1RM	60%	60%	70%	70%	60%	60%	70%	70%
Full squat training (FST)	3 × 8	4 × 8	4 × 10	5 × 12	3 × 8	4 × 10	5 × 12	5 × 12
Half squat training (HST)	3 × 8	4 × 8	4 × 10	5 × 12	3 × 8	4 × 10	5 × 12	5 × 12

**Table 2:** Descriptive characteristics of the sample.

	Training Group	N	Mean	Std. Deviation
Age (years)	FST	14	13.96	0.95
	HST	14	13.79	0.89
Height (cm)	FST	14	166.79	13.06
	HST	14	165.37	9.66
Sitting height (cm)	FST	14	84.35	6.13
	HST	14	80.74	5.38
Body mass (kg)	FST	14	59.19	8.66
	HST	14	55.28	8.74
Body fat (%)	FST	14	15.02	2.61
	HST	14	13.66	2.80
PHV (years)	FST	14	0.48	1.11
	HST	14	0.41	0.84
APHV (years)	FST	14	13.62	0.45
	HST	14	13.81	0.33

Notes for the table: APHV: age at peak height velocity; FST: full squat training; HST: half squat training; PHV: distance to peak-height velocity.

**Table 3:** Anthropometry and body composition results.

Measure	Training Group	Time	Mean	Std. Dev	95% Confidence Interval		Significance (Time)	Effect Size
					Lower	Upper		
Body mass (kg)	FST	Pre-intervention	59.19	8.66	54.23	64.14	0.403	0.03
		Post-intervention	59.43	9.36	54.49	64.37		
	HST	Pre-intervention	55.28	8.74	50.33	60.23	<0.001	0.18
		Post-intervention	56.93	9.24	51.99	61.87		
Body fat (%)	FST	Pre-intervention	15.02	2.61	13.53	16.51	<0.001	2.05
		Post-intervention	9.62^(0.032)^	2.80	8.31	10.92		
	HST	Pre-intervention	13.66	1.93	12.17	15.15	<0.001	0.72
		Post-intervention	11.66	2.75	10.35	12.96		
Muscle volume of the thigh (cm^3^)	FST	Pre-intervention	3.19	0.78	2.82	3.56	<0.001	2.26
		Post-intervention	4.92	0.55	4.56	5.28		
	HST	Pre-intervention	3.07	0.74	2.70	3.44	<0.001	3.03
		Post-intervention	4.79	0.57	4.43	5.15		
Muscle volume of the calf (cm^3^)	FST	Pre-intervention	1.51	0.28	1.36	1.65	<0.001	4.54
		Post-intervention	3.62	0.23	3.41	3.84		
	HST	Pre-intervention	1.39	0.47	1.24	1.53	<0.001	7.89
		Post-intervention	3.52	0.30	3.30	3.73		
Muscle volume of the leg (cm^3^)	FST	Pre-intervention	4.69	1.04	4.20	5.18	<0.001	2.04
		Post-intervention	6.80	0.72	6.32	7.28		
	HST	Pre-intervention	4.46	1.01	3.97	4.95	<0.001	3.04
		Post-intervention	6.65	0.72	6.17	7.13		
Cross-sectional area 1/2 thigh (cm^2^)	FST	Pre-intervention	119.88	30.82	105.42	134.35	<0.001	0.35
		Post-intervention	130.95	20.91	116.48	145.41		
	HST	Pre-intervention	109.99	30.90	95.52	124.46	<0.001	0.46
		Post-intervention	121.04	20.79	106.57	135.50		
Cross-sectional area max thigh (cm^2^)	FST	Pre-intervention	159.89	48.04	139.03	180.75	<0.001	0.43
		Post-intervention	180.86	24.02	159.98	201.73		
	HST	Pre-intervention	151.91	48.06	131.05	172.78	<0.001	0.88
		Post-intervention	173.00	24.04	152.13	193.88		

Notes for the table: Significant between-group differences are presented in superscript parentheses. FST: full squat training; HST: half squat training.

**Table 4:** Neuromuscular performance results.

Measure	Group	Time	Mean	Std. Dev	95% Confidence Interval		Significance (Time)	Effect Size
					Lower	Upper		
Mean propulsive velocity at 50%1RM (m/s)	FST	Pre-intervention	0.54	0.03	0.52	0.55	<0.001	2.47
		Post-intervention	0.64^(<0.001)^	0.03	0.61	0.67		
	HST	Pre-intervention	0.52	0.05	0.50	0.53	0.014	0.92
		Post-intervention	0.56	0.06	0.53	0.59		
Mean propulsive velocity at 45%1RM (m/s)	FST	Pre-intervention	0.62	0.03	0.61	0.64	0.008	4.07
		Post-intervention	0.73^(<0.001)^	0.03	0.71	0.75		
	HST	Pre-intervention	0.64	0.03	0.63	0.66	0.008	0.82
		Post-intervention	0.67	0.04	0.65	0.69		

Notes for the table: Significant between-group differences are presented in superscript parentheses. FST: full squat training; HST: half squat training.

## Data Availability

The data presented in this study are available on request from the corresponding author.
